# Depression status and functional outcome of patients with ischemic stroke and the impact on caregivers living in Chengdu: a cross-sectional study

**DOI:** 10.3389/fpsyt.2023.1166273

**Published:** 2023-07-04

**Authors:** Lanying He, Jian Wang, Feng Wang, Lu Wang, Yinglin Liu, Fanfan Zhou, Fan Xu

**Affiliations:** ^1^Department of Neurology, The Second People’s Hospital of Chengdu, Chengdu, China; ^2^Department of Public Health, Chengdu Medical College, Chengdu, Sichuan, China

**Keywords:** stroke survivors, caregiver, depression, mental health, caregiver burden, Zarit burden interview

## Abstract

**Objectives:**

To investigate the associations between risk factors and depression symptoms in ischemic stroke (IS) survivors and the effect of IS survivors’ depression status and functional outcomes on caregiver burden in Chengdu, China.

**Methods:**

In this cross-sectional study, we recruited a convenience sample of patients with IS and paired caregivers living in Chengdu from February 2022 to May 2022. Depression symptoms were assessed using the 17-item Chinese Hamilton Depression Rating Scale, the social support of patients was assessed using the perceived social support scale (PSSS), caregiver burden was assessed using the Zarit burden interview (ZBI). Multivariable logistic regression analysis was used to analyze the data between risk factors and depression symptoms, and multiple linear regression models were constructed to examine the depression symptoms and functional outcomes of stroke survivors, and caregiver burden.

**Results:**

In total, 966 IS survivors and paired caregivers were included in this study. Among IS survivors, 35.51% (343/966) experienced depression. Age [adjusted odds ratio (aOR), 1.02; 95% confidence interval (CI), 1.00–1.04; *p* = 0.036], the National Institutes of Health Stroke Scale (NIHSS) score (aOR, 1.57; 95% CI, 1.47–1.68; *p* < 0.001), and PSSS score (aOR, 0.86; 95% CI, 0.84–0.89; *p* < 0.001) were associated with an increased risk of depression. The NIHSS score (*b* = 2.57, *p* < 0.001), patients’ depression status (*b* = 2.54, *p* < 0.001), duration of care (*b* = 0.359, *p* = 0.006), and social support of caregivers (*b* = −0.894, *p* = 0.038) were significantly associated with the ZBI score.

**Conclusion:**

The PSSS score was a major risk factor for the development of depression in IS survivors, and patients’ depression status and severe functional deficits had a negative impact on the ZBI score of the main caregivers. Social support can reduce the ZBI score.

## Introduction

Stroke is the leading cause of long-term disability in the Chinese population (1). Although there have been significant advances in the treatment of ischemic stroke (IS), including recanalization, the incidence of stroke has declined recently, but the number of strokes continues to increase with the accelerated aging population and longer life expectancy; many IS survivors still have moderate to severe disabilities after treatment (2–4). In Asia, the incidence of stroke is approximately 116 to 483 per 100,000 people per year (5), and according to the latest statistics released by the National Health Commission, the total number of stroke cases in China exceeded 28 million in 2021.

Depression, anxiety, and mental sequelae are very common among IS survivors, with the prevalence of depression ranging from 14% to 50% (6–8). Poor mental health has a negative impact on neurological rehabilitation and quality of life. Previous studies have shown that social support has positive effects on functional recovery and mental health after stroke (9–12). Social support comes from family members, colleagues, friends, and community groups. Social support is divided into objective support, which is the actual help from others, and perceived social support, which is the perceived support resulting from confidence in support for physical or emotional needs. Some evidence suggested that there is a relationship between objective social support and mental health and physical prognosis (13, 14); however, previous studies that aimed to investigate the association between perceived social support and mental health are limited. Because of the limited evidence and differences in cultures and health systems across countries, it is necessary to better understand the relationship between post-IS mental health and perceived social support in China.

Depending on the severity of the functional impairments, IS survivors may require caregivers to assist with their daily needs and care for their physical, cognitive, and mental health (15). Over the years, literature has emphasized the interaction between patients and caregivers (16). Some studies have shown that caregivers’ well-being can affect patient clinical outcomes (17, 18). For example, caregivers with poor mental health are more likely to result in patients entering care facilities (19, 20).

A concept related to a patient caregiver is caregiver burden. It is defined as the physical and emotional response of the caregiver during the course of patient care; studies have shown that 25%–54% of caregivers experience significant caregiving burden (8, 21–23).

To date, some studies have explored the association between caregiver burden and the severity of patients’ functional deficits (24, 25). Yet, few studies have described the impact of depression symptoms on caregiver burden.

Therefore, to address this research gap, we investigated the association between perceived social support and depression status in IS survivors, and the effect of patients’ depression status and functional outcome on caregiver burden.

## Materials and methods

### Study design and population

This cross-sectional study was conducted between February 2022 and May 2022. Fifteen districts and counties in Chengdu, China were used as research sites. First, the streets/towns in each district or county were numbered. Second, we used the random number table method to scientifically select random samples, and two communities/administrative villages were selected from each street/township. Finally, two residential areas/villages were extracted from each community/administrative village.

IS was identified based on patients’ medical history or discharge certificates. The National Institutes of Health Stroke Scale (NIHSS) was used to assess stroke severity. All IS survivors and their caregivers were paired. The caregiver was a family member who lived with the patient and took care of the patient’s physical and daily activities, including communication and emotional support.

### Inclusion and exclusion criteria

IS survivors and their paired caregivers were included in this study. After being discharged from general hospitals, most IS patients underwent short rehabilitation training in community hospitals and then returned to their families. The primary caregiver was defined as the person taking care of the patient’s daily activities, including assisting the patient with walking, turning over in bed, eating, defecating, and bathing, as well as providing emotional support.

Inclusion criteria for caregivers were (1) age ≥18 years, (2) family member, and (3) duration of care ≥6 months and ≥8 h/day. The exclusion criteria were (1) severe renal failure (estimated glomerular filtration rate <30 mL/min/1.73 m^2^), severe lung disease, active malignancy, and severe heart disease (cardiac function >grade II); (3) dyslexia, deafness, or mental impairment and inability to complete the survey; (4) history of psychosis; and (5) alcohol or drug abuse. Caregivers’ physical and mental health was obtained from their medical history base on consulting caregivers or reviewing outpatient/discharge certificates.

The inclusion criteria for patients were as follows: (1) modified Rankin scale score of 0 before stroke and (2) living at home. The exclusion criteria for patients were as follows: (1) neurological symptoms unrelated to IS and (2) inability to complete the scale because of deafness, mental impairment, aphasia, and severe dementia.

### Data collection

All data were collected online and automatically entered into a spreadsheet. Two trained neurologists evaluated all scale scores.

Patient demographics and clinical characteristics including hypertension, diabetes mellitus, hyperlipidemia, smoking history, medication use, and patient income were collected. Caregivers’ demographics including age, sex, education level, chronic diseases, duration of care, and Zarit burden interview (ZBI) score were collected.

Chronic diseases of caregivers included hypertension, diabetes, mild to moderate chronic obstructive pulmonary disease, mild to moderate liver dysfunction, mild to moderate renal dysfunction, mild to moderate asthma, mild to moderate stroke, mild to moderate epilepsy, mild to moderate multiple sclerosis, mild to moderate Alzheimer disease, mild to moderate Parkinson disease, and history of cancer.

Patients’ social support was evaluated using the perceived social support scale (PSSS), which was designed by Zimet et al. in 1990 and first translated into Chinese in 2001. A previous study showed that the PSSS has good validity and reliability (26). The scale consists of 12 items designed to measure an individual’s perceptions of the support received from family members and other individuals, including friends, leaders, relatives, and colleagues. Each item is scored on a scale of 0–7 (1 = strongly disagree, 7 = strongly agree). The overall score reflects an individual’s overall social support, with higher scores indicating higher levels of perceived support.

The 17-item Chinese Hamilton depression rating scale (HDRS) was used to assess the severity of depression (27, 28); each item is scored on a scale of 0–4. A total score of 0–6 is considered to indicate no depression, 7–16 indicates mild depression, 17–24 indicates moderate depression, and >24 indicates severe depression. A definite depressive state was defined as a 17-item Chinese HDRS score >17. The validity and reliability of the 17-item Chinese HDRS have been verified in a previous study (28).

Caregiver burden was evaluated using the ZBI, a self-reported questionnaire that reflects caregivers’ negative views on caregiving. A previous study showed that the shorter 12-item version of the ZBI had good validity and reliability (Cronbach alpha = 0.93), with responses ranging from 0 to 4: 0 = never, 1 = rarely, 2 = sometimes, 3 = quite frequently, and 4 = nearly always (29).

### Statistical analysis

Data are presented as numbers (%) or means (±standard deviation). The Pearson *χ*^2^ test was used to analyze categorical variables. The student *t*-test was used to compare normally distributed variables between groups, whereas the Mann–Whitney *U* test was used to compare non-normally distributed variables between groups. Multivariable logistic regression analysis was used to analyze data between risk factors and depression symptoms, and multiple linear regression models were constructed to examine stroke survivors’ depression symptoms, functional outcomes, and caregiver burden. The results are expressed as adjusted odds ratios (aORs) with corresponding 95% confidence intervals (CIs). The data were analyzed using SPSS 22 software (IBM Corp.). Statistical significance was set at *p* < 0.05.

## Results

### Patient characteristics

**Figure 1:**
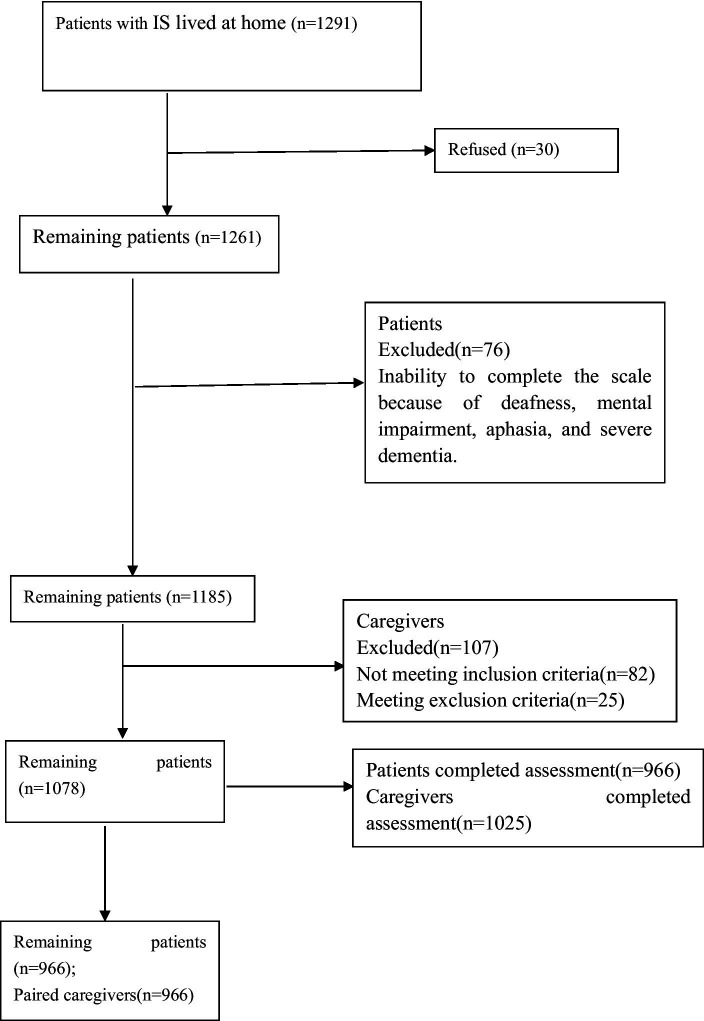
Patient’s and caregiver’s flowchart.

Of the 1,078 eligible participants, 966 with IS participated in the study, resulting in a response rate of 89.61% (Figure 1). Patients’ mean age was 67.58 ± 10.62 years, 497 (51.45%) were female, 640 (66.25%) patients had hypertension, 409 (42.34%) had diabetes, 559 (57.84%) had hyperlipidemia, 245 (25.36%) smoked, 288 (29.81%) drank alcohol, 116 (12.01%) had gastric tubes, and 164 (16.98%) had urinary catheter. The mean NIHSS score was 9.96 ± 4.18. Only 653 (67.60%) patients were taking antiplatelet aggregation drugs, and 519 (53.73%) were taking lipid-lowering drugs. The mean PSSS score was 68.27 ± 8.42.

### Risk factors for depression among is survivors

The baseline characteristics of patients in the non-depression and depression groups were compared (Table 1). At baseline, the group of patients with depression status had older age [69.04 ± 10.28 versus (vs.) 66.78 ± 10.72, *p* < 0.001], higher NIHSS score (13.57 ± 4.80 vs. 9.71 ± 4.00, *p* < 0.001), and higher percentage of women than those without depression [53.06% vs. 46.07%; odds ratio (OR), 1.32; 95% CI, 1.02–1.72; *p* = 0.037]. Compared with the non-depression group, the depression group had a significantly lower PSSS score (62.32 ± 7.77 vs. 71.55 ± 6.82, *p* < 0.001).

**Table 1:** The demographic characteristics of patients in no PSD status and PSD status groups.

	No PSD status group (623)	PSD status group (343)	OR (95%CI)	*p* ^a^
Age, y (mean ± SD)	66.78 ± 10.72	69.04 ± 10.28		**<0.001**
NIHSS, (mean ± SD)	9.71 ± 4.00	13.57 ± 4.80		**<0.001**
Time since stroke, y (mean ± SD)	3.14 ± 1.28	3.26 ± 1.40		0.227
Females, *n* (%)	287 (46.07)	182 (53.06)	1.32 (1.02–1.72)	**0.037**
BMI ≥24 kg/m^2^, *n* (%)	184 (29.53)	89 (25.94)	0.84 (0.62–1.13)	0.236
Smoking, *n* (%)	151 (24.34)	94 (27.41)	1.18 (0.87–1.59)	0.279
Alcohol, *n* (%)	188 (30.18)	100 (29.15)	0.95 (0.71–1.27)	0.740
Diabetes, *n* (%)	267 (42.86)	142 (41.40)	0.94 (0.72–1.23)	0.661
Hyperlipidemia, *n* (%)	365 (58.59)	194 (56.56)	0.92 (0.71–1.20)	0.541
Hypertension, *n* (%)	414 (66.45)	226 (65.89)	0.98 (0.74–1.29)	0.859
Nasogastric tube feeding, *n* (%)	76 (12.20)	40 (11.66)	0.95 (0.63–1.43)	0.806
Indwelling urinary catheter, *n* (%)	101 (16.21)	63 (18.36)	1.16 (0.82–1.64)	0.393
Medications use				
Antiplatelet, *n* (%)	426 (68.38)	227 (66.18)	0.91 (0.68–1.20)	0.485
Antihypertensive, *n* (%)	345 (55.38)	179 (52.19)	0.88 (0.68–1.45)	0.341
Lipid-lowering medications, *n* (%)	334 (53.61)	185 (53.94)	1.01 (0.78–1.32)	0.923
Patient’s income, Chinese Yuan				
Low personal income (≤5,000 CNY/month), *n* (%)	308 (49.44)	192 (55.98)	1.300 (1.00–1.70)	0.052
PSSS, (mean ± SD)	71.55 ± 6.82	62.32 ± 7.77		**<0.001**

a Comparison between no PSD status and PSD status groups. Continuous variables are expressed as mean ± standard deviation (SD). Baseline characteristics were compared between the 2 subgroups by univariate analysis using Pearson *χ*^2^, distributions of continuous variables were determined by the Kolmogorov–Smirnov test, Mann–Whitney two sample test was applied in case of non-normal distributions. Bold indicates *p*-values less than 0.05.

### Multivariable models on the association between risk factors and patients’ depression status

In the unadjusted models, older age (aOR, 1.02; 95% CI, 1.01–1.03; *p* = 0.02), higher NIHSS score (aOR, 1.66; 95% CI, 1.56–1.77; *p* < 0.001), higher PSSS score (aOR, 0.84; 95% CI, 0.82–0.86; *p* = 0.02), and female (aOR, 1.32; 95% CI, 1.02–1.72; *p* = 0.038) were associated with an increased risk of depression in patients with IS.

To further analyze the association between risk factors and the depression status of patients with IS, the factors associated with depression status in the univariate analyses (*p* < 0.20) were entered into the multivariate logistic regression analysis, which was adjusted for age, baseline NIHSS score, PSSS score, sex, and low household income. Older age (aOR, 1.02; 95% CI, 1.00–1.04; *p* = 0.036), higher NIHSS score (aOR, 1.57; 95% CI, 1.47–1.68; *p* < 0.001), and PSSS score (aOR, 0.86; 95% CI, 0.84–0.89; *p* < 0.001)were associated with an increased risk of depression in patients with IS (Table 2).

**Table 2:** Multivariable models showing association between baseline risk factors and depression.

Risk factors	OR (95% CI)	*p* ^a^
Age	1.02 (1.00–1.04)	**0.036**
NIHSS	1.57 (1.47–1.68)	**<0.001**
PSSS	0.86 (0.84–0.89)	**<0.001**

a Multivariable adjusted for age, baseline NIHSS, PSSS, gender, low household income. Bold indicates *p*-values less than 0.05.

### Characteristics of the main caregivers

In total, 966 paired main caregivers were included; their mean age was 55.47 ± 10.09 years, and 305 (31.57%) were female. The mean duration of care was 3.10 ± 1.39 years, and mean ZBI score was 30.15 ± 11.67. Overall, 188 (19.46%) main caregivers had social support, 396 (40.99%) had high school and above education, and 600 (62.11%) were a spouse. Among patients, 307 (31.78%) were self-financed, 399 (41.30%) had ≥2 chronic diseases, and 305 (31.57%) drank alcohol. There were 528 (60.87%) main caregivers with a personal income of <5,000/month (CNY) (Table 3).

**Table 3:** The baseline characteristics of caregivers.

Baseline characteristics	*N* = 966
Caregivers’ age, y (mean ± SD)	55.47 ± 10.09
NIHSS of patients, (mean ± SD)	9.96 ± 4.18
Duration of care time, y (mean ± SD)	3.10 ± 1.39
ZBI (mean ± SD)	30.15 ± 11.67
Females, *n* (%)	305 (31.57)
Social supports of caregivers, *n* (%)	188 (19.46)
Patients with depression, *n* (%)	343 (35.51)
Education (high school and above), *n* (%)	396 (40.99)
Relationship to the patient	
Spouse, *n* (%)	600 (62.11)
Other family member, *n* (%)	366 (37.89)
Self-finance, *n* (%)	307 (31.78)
Personal income of main caregivers, Chinese Yuan (CNY) <5,000/month, *n* (%)	528 (60.87)
BMI ≥24 kg/ m^2^, *n* (%)	281 (29.09)
Alcohol, *n* (%)	305 (31.57)
Chronic disease ≥2, *n* (%)	399 (41.30)

### Multivariate linear regression models on the association between risk factors and ZBI scores

According to the HDRS results, 343 patients had depression. The average ZBI score of main family caregivers was 38.04 ± 12.14 among those caring for patients with depression; that value was significantly higher than the average ZBI score of caregivers caring for patients without depression (25.81 ± 8.77, *p* < 0.001).

Multivariate linear regression analysis, which was adjusted for age, sex, patients’ NIHSS score, duration of care, social support of caregivers, education (high school and above), depression status, body mass index ≥24 kg/m^2^, self-financed patient, personal income of main caregivers, current alcohol use, and ≥2 chronic diseases, was performed to examine the independent associations of the ZBI score. The NIHSS score of patients (*b* = 2.57, *p* < 0.001), depression status (*b* = 2.54, *p* < 0.001), duration of care (*b* = 0.359, *p* = 0.006), and social support of caregivers (*b* = −0.894, *p* = 0.038) were significantly associated with the ZBI score (Table 4).

**Table 4:** Multiple linear regression analysis for factors significantly associated with ZBI.

Factors	ZBI		
	Coefficient (*b*)	Standard error	*p*^a^-value
NIHSS of patients	2.57	0.922	<0.001
Depression status of patients	2.54	0.104	<0.001
Duration of care time	0.359	0.043	0.006
Social supports of caregivers	−0.894	−0.036	0.038

Bold indicates *p*-values less than 0.05.

a Multivariable linear regression adjusted for adjusted for age, gender, NIHSS of patients, duration of care time, social supports of caregivers, education (high school and above), patients with depression status, BMI ≥24 kg/m^2^, self-finance of patients, personal income of main caregivers, current alcohol, chronic disease ≥2.

## Discussion

In this study of IS stroke survivors and paired main caregivers, we found a high incidence of depression symptoms in patients with IS; 343 (35.51%) patients had an HDRS score >17. A lower PSSS score was associated with an increased risk of depression, independent of other confounding factors. The data showed positive associations between the ZBI score and depression status and the severity of IS in stroke survivors.

Stroke is the leading cause of disability (30–32). Previous studies have revealed that most IS survivors leave hospitals with varying degrees of disability; they are often accompanied by limb hemiplegia and other dysfunctions (33–35). In the presence of physical impairment, social support may affect a person’s ability to overcome environmental challenges outside the home, and it may also be a protective factor against depressive symptoms after stroke. Care from caregivers often focuses on daily living and transfer, and little on community reintegration and participation of IS patients (15, 36). Perceived social support plays an important role in the mental health of stroke survivors. In this study, we found that lower perceived social support after IS was associated with a higher risk of depression. These findings suggest that perceived social support may protect against mental disorders following IS. The use of social support in prevention and treatment can prevent mental health symptoms after stroke.

Although previous studies have shown that the prevalence of depression was higher in younger IS stroke survivors than in older ones (37, 38), our study showed that older IS stroke survivors were more vulnerable and at a higher risk of depression than younger ones. This difference might be due to differences in the research methods used. A systematic review of studies on the relationship between age and post-stroke depression showed that population origin (hospitalized versus community-resident survivors) and timing of stroke onset were factors that might contribute to different outcomes (39). The more serious the sequelae of stroke, the higher the degree of patient dependence, the higher the NIHSS score, and the more need for assistive aids. The use of these assistive aids was found to increase the risk of depression.

In the present study, the results showed that a lower PSSS score had a negative effect on the depressive status of patients. Additionally, there was a strong association between the severity of IS and caregiver burden, and depressive symptoms might increase this association, as well as a direct negative effect on caregiver burden. In the general population, the mental state of the caregiver is associated with caregiver burden, and more importantly, caregiver burden is significant in predicting caregiver depression (40). This study further confirmed that the depression status of patients is associated with higher ZBI scores.

Support measures should be provided to both IS survivors and caregivers. For example, outpatient care services and psychological assistance, especially systemic therapy, psychoeducation, and self-help groups, could alleviate social tension among family members when caregivers are under high stress. Social support has been reported to be associated with lower levels of psychological burden and better adjustment among caregivers (41, 42). Herein, there was a positive association between the duration of care and ZBI score; the longer the care time, the higher the ZBI score of the caregiver. Previous studies have also shown that a higher care burden is more likely to appear with longer care time (43, 44). This may be because the long day of care takes too much of the caregiver’s personal time, minimizing their work, social, and entertainment time.

We also investigated the association between perceived social support and depression status in IS survivors and the effect of patients’ depression status and functional outcomes on caregiver burden. The study’s results demonstrated that patient age, NIHSS score, and PSSS score were associated with depression status among IS survivors, and the depression status and NIHSS score of patients, and lower social support had a negative effect on caregiver burden. Therefore, it may be suggested that any support, such as social and psychological support, relieves depression in IS survivors and caregivers’ care burden and will be helpful in improving the quality of life of IS survivors and caregivers.

### Limitations

This study has some limitations. First, this was a cross-sectional study, and the results can only represent the current situation of stroke survivors and caregivers in Chengdu. Second, depression symptoms are dynamic and were evaluated at only a single time point. Third, we did not collect data on patients’ use of antidepressants. Fourth, the 17-item Chinese HDRS score is just a screening tool. We used the17-item Chinese HDRS to assess whether the patients had symptoms of depression, but it should not be used to diagnose depression. Lastly, we did not analyze the effect of different levels of depression on caregivers’ ZBI score, which might have influenced the results.

## Conclusion

In conclusion, patient age, NIHSS score, and PSSS score were major risk factors for the development of depression in IS survivors. The depression status and functional deficits of patients had a negative impact on the ZBI score of the main caregivers, but social support can reduce the ZBI score. These findings further confirm that community doctors or social groups should provide social support, family nursing guidance, and psychological counseling to improve the mental health of family caregivers.

## Data availability statement

The original contributions presented in the study are included in the article/supplementary material, further inquiries can be directed to the corresponding author.

## Ethics statement

The studies involving human participants were reviewed and approved by Medical and Health Research Ethics Committee in Second people’s Hospital of Chengdu. The patients/participants provided their written informed consent to participate in this study. Written informed consent was obtained from the individual(s) for the publication of any potentially identifiable images or data included in this article. 

## Author contributions

LH was responsible for the concept and design of the study, data collection and analysis, the first draft of the paper, and further manuscript. JW was responsible for concept and design of the study, the data analysis and interpretation. FW was responsible for the data analysis. FZ, LW, and YL were responsible for data collection. FX was responsible for overseeing the concept and design of the study. All authors contributed to the article and approved the submitted version.

## Funding

This work was funded by the Health and Family Planning Commission of Chengdu (2015009), the Sichuan Medical Association (Q14011), the Chengdu Science and Technology Bureau Focuses on Research and Development Support Plan (2019-YF09-00097-SN) the Chengdu Medical College Natural Science Foundation (CYZ18-33, CYZ19-33), the Sichuan Provincial Education Department (17ZA0134), the Sichuan Medical Association (S18023). The funding body did not participate in designing the study or writing the manuscript. The study protocol had undergone peer-review process by the funding body.

## Conflict of interest

The authors declare that the research was conducted in the absence of any commercial or financial relationships that could be construed as a potential conflict of interest.

## Publisher’s note

All claims expressed in this article are solely those of the authors and do not necessarily represent those of their affiliated organizations, or those of the publisher, the editors and the reviewers. Any product that may be evaluated in this article, or claim that may be made by its manufacturer, is not guaranteed or endorsed by the publisher.
